# Investigating the Mechanism of Unilateral Cross Incompatibility in Longan (*Dimocarpus longan* Lour.) Cultivars (Yiduo × Shixia)

**DOI:** 10.3389/fpls.2021.821147

**Published:** 2022-02-11

**Authors:** Jing Wang, Ji Chen, Shilian Huang, Dongmei Han, Jianguang Li, Dongliang Guo

**Affiliations:** ^1^Institute of Fruit Tree Research, Guangdong Academy of Agricultural Sciences, Guangzhou, China; ^2^Key Laboratory of South Subtropical Fruit Biology and Genetic Resource Utilization, Guangdong Provincial Key Laboratory of Tropical and Subtropical Fruit Tree Research, Ministry of Agriculture and Rural Affairs, Guangzhou, China; ^3^College of Horticulture, South China Agricultural University, Guangzhou, China

**Keywords:** Chinese longan, intraspecific cross incompatibility, jasmonic acid metabolism, plant-hormone, gene expression

## Abstract

Longan (*Dimocarpus longan* Lour.) is an important subtropical fruit tree in China. Nearly 90% of longan fruit imports from Thailand are from the cultivar Yiduo. However, we have observed that there exists a unilateral cross incompatibility (UCI) when Yiduo is used as a female parent and Shixia (a famous Chinese cultivar) as a male parent. Here, we performed a comparative transcriptome analysis coupled with microscopy of pistils from two reciprocal pollination combinations [Shixia♂ × Yiduo♀(SY) and Yiduo♀ × Shixia♂(YS)] 4, 8, 12, and 24 h after pollination. We also explored endogenous jasmonic acid (JA) and jasmonyl isoleucine (JA-Ile) levels in pistils of the crosses. The microscopic observations showed that the UCI was sporophytic. The endogenous JA and JA-Ile levels were higher in YS than in SY at the studied time points. We found 7,251 differentially expressed genes from the transcriptome analysis. Our results highlighted that genes associated with JA biosynthesis and signaling, pollen tube growth, cell wall modification, starch and sucrose biosynthesis, and protein processing in endoplasmic reticulum pathways were differentially regulated between SY and YS. We discussed transcriptomic changes in the above-mentioned pathways regarding the observed microscopic and/or endogenous hormone levels. This is the first report on the elaboration of transcriptomic changes in longan reciprocal pollination combination showing UCI. The results presented here will enable the longan breeding community to better understand the mechanisms of UCI.

## Introduction

Longan (*Dimocarpus longan* Lour.) belongs to the *Sapindaceae* family. It is one of the most important subtropical fruit trees indigenous in China. Within China, major longan-producing areas are distributed between 18–31.16°N latitude and 100°44′–122°E longitude, Guanxi, Guangdong, and Fujian are the major production areas (Qiu, 2012). The fruit is rich in nutrients and has been regarded as “precious tonic” since ancient times. It is believed that longan originated in China, since it has been cultivated for thousands of years (Subhadrabandhu and Yapwattanaphun, 2001). Recently, China has been importing large volumes of longan from nearby countries, i.e., Thailand, Vietnam, and Cambodia (Hasachoo and Kalaya, 2013). In 2020, China imported 72.67 MMT of longan fruits^1^. The increasing imports are impacting local production. There are more than 400 longan cultivars registered in China. The major cultivars are Shixia, Chuliang, Dawuyuan, and Fuyan. These cultivars are cultivated in different regions depending on their traits and suitability to the local climate. Shixia is the most cultivated cultivar in Guangdong province. However, its production is lower than those that are grown in Thailand. A major example is the Thai longan cultivar “E-Daw,” which is known as Yiduo in China (Choo, 2000). Yiduo is a tropical ecotype that was introduced in China by Thailand. Its yield is relatively higher than that the Shixia or other cultivars in China. Yiduo production per mu can reach 2,000–3,000 kg in Thailand. After its introduction in China, its production has declined and is mainly dependent on self-pollination to bear fruits. Yiduo has several useful traits such as easy flowering, coarse and large fruits, vigorous growth, high edible rate of fruit, and high yield and quality of fruits, which have attracted the attention of Chinese longan breeders to include it in Chinese longan breeding programs. Our research group has tried to improve Yiduo using local cultivars as male parent by hybridization. However, in three successive years, we have noticed that there exists a unilateral cross incompatibility (UCI) when Yiduo is used as a female parent and Shixia as a male parent, i.e., Yiduo♀ × Shixia♂(YS). Our earlier data showed a fruit setting rate of 22.4% and zero percent in SY and YS, respectively. Understanding this reproductive barrier is a prime target for longan breeders to uplift the quality of longan in China.

Unilateral cross incompatibility has been reported in multiple plant species, e.g., capsicum (Onus and Pickersgill, 2004), faba bean (Abdalla, 1977), and field mustard (Takada et al., 2017), and is defined as an intraspecific relationship in which pollinations are only compatible in one direction (Tovar-Méndez et al., 2014). In *Solanum* species, UCI has been reported because of rejection of pollen from self-compatible species on the pistil of self-incompatible species (Tovar-Méndez et al., 2014). In tomato, this UCI was linked to the absence of S-RNAse. In brassica species, the self-incompatibility is regulated by an S-haplotype-specific interaction between S-receptor kinase (stigma-specific expression) and S locus protein 11 (tapetum-cell-specific expression) (Takada et al., 2017). Generally, it is considered that pre-fertilization/pre-zygotic/post-pollination barriers can be responsible for such UCI that could be due to failure of pollen germination or pollen growth in pistil (De Nettancourt, 2001). Pollen tube growth is different from that of other plant cells, as its growth is mainly restricted to the tip region (Mascarenhas, 1993). Under conditions of pollen competition, only fast-growing pollen tubes can accomplish effective fertilization. Pollen tube growth varies in different species (Stone et al., 2004), and certain factors such as calcium and potassium ion concentration (Caser, 2017), biosynthesis of cell wall polymers (Mollet et al., 2013), and availability and concentration of sucrose/carbohydrates (O’Kelley, 1955; Stadler et al., 1999) can contribute to pollen tube growth. These factors are controlled by a large number of genes belonging to different biosynthetic processes, i.e., plant hormone signaling, cell enlargement, metabolism-related pathways, cell wall biosynthesis and rearrangement, and protein processing (Becker and Feijó, 2007). An understanding of the expression of genes in specific pathways would enable us to explore the possible reasons for UCI in YS. Recent advancements in transcriptome sequencing technology have enabled researchers to understand the molecular mechanisms underlying cross incompatibility in maize (Wang et al., 2018), self-incompatibility in lemon (Zhang et al., 2015), tea (Zhang et al., 2016), and oilseed camelia (He et al., 2020), and pollen tube development in olive (Iaria et al., 2016). A similar approach would help us to explore transcriptomic signatures by comparing different pollination combinations in Yiduo and Shixia longan trees.

Methods to overcome UCI include genetic rescue (Holmes et al., 2008), use of hormone application together with irradiated pollen and mixed pollination, grafted ovary method, cut-style method, e.g., *Lycopersicon* (*Lycopersicum esculentum* × *Lycopersicum peruvianum*) (Majid, 1964), Tulipa (Van Creij et al., 1997), and lily (Van Creij et al., 2000). Since pollen germination and pollen tube growth are development-related processes, the role of particular hormones cannot be negated. For example, it has been reported that pollen germination and pollen tube growth in apricot and *Pinus nigra* are influenced by methyl jasmonate (MeJA) (Muradoğlu et al., 2010; Çetinbaş-Genç and Vardar, 2020). Similarly, higher endogenous jasmonic acid (JA) levels have also been related to limited pollen germination in Arabidopsis (Ju et al., 2016).

In this study, we designed a reciprocal pollination combination of these two cultivars and explored transcriptomic signatures in pistil in different hours after pollination (HAP) that could be associated with UCI. We discuss the possibilities that differences in pollen tube growth can be linked with UCI. Furthermore, we tried to understand and explain if there are changes in the endogenous levels of JA and its derivative MeJA. We discussed the possible roles of pollen tube-related genes, phytohormone signaling, starch and sucrose biosynthesis, phenylpropanoid biosynthesis, JA metabolism and signaling, and protein processing in endoplasmic reticulum pathways in UCI.

## Materials and Methods

### Plant Materials

**FIGURE 1:**
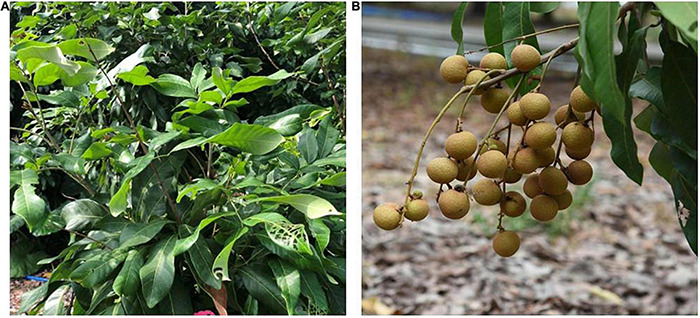
Phenotype of the two reciprocal crosses between Yiduo (Y) and Shixia (S). **(A)** Yiduo♀ × Shixia♂(YS) showing no fruiting. **(B)** Shixia♀ × Yiduo♂(SY) showing fruiting.

Three plants of two *Dimocarpus longan* Lour. Cultivars, i.e., Shixia and Yiduo, were selected from the Longan Resource Nursery of Guangzhou City, Guangzhou, China. Care was taken while selecting the longan trees, and it was ensured that the selected trees were of the same growth and age (10 years old). The flower spikes present on different sides of the trees were chosen on 28 March 2020 for pollination and covered with waterproof sulfuric acid bags (40 cm^2^ × 30 cm^2^). Artificial pollinations were carried out in two reciprocal combinations, i.e., combination I: Yiduo♀ × Shixia♂(YS) and combination II: Shixia♀ × Yiduo♂(SY) (Figure 1). The pollinations were carried out as reported earlier (McConchie et al., 1994; Pham, 2012). Each pollination combination was marked for identification. Briefly, on 31 March 2020 at 8:30 a.m., the bags were removed at the same time from the flowers chosen as female, and pistils were removed before pollination and immediately stored in liquid nitrogen; each variety had three replicates. After this, artificial pollination was carried out using the male flowers bloomed in the same morning, and the pollinated flowers were bagged. Pistils were collected 0, 1, 4, 8, 12, and 24 h after pollination (HAP), quickly frozen in liquid nitrogen, and stored at −70° or in a Kano fixing solution. The stored samples were then used for detection of endogenous phytohormone levels (0, 4, 8, 12, and 24 HAP), transcriptome sequencing, and *in vitro* study on pollen germination and pollen tube growth (0, 4, 8, 12, and 24 HAP).

### Determination of Phytohormone Levels

An AB Sciex QTRAP6500 LC-MS/MS system was used to detect the content of phytohormones before and after pollination in the different pollination combinations reported earlier (Xiao et al., 2018; López-Cristoffanini et al., 2019).

### Study on Pollen Germination and Pollen Tube Growth

Pollen *in situ* germination and pollen tube growth in the style and ovary were observed as described by Xie et al. (2019), with slight modifications. Shixia pistils were pollinated with Yiduo pollens, and Yiduo pistils were pollinated with Shixia pollens; then, pistils were picked 0, 4, 8, 12, and 24 HAP separately. Isolated pistils were then fixed in Carnoy’s fixative solution for 1 day. The pistils were then cut dorsally after gradient rehydration and softened with 4% NaOH for 12 h. The cut and softened pistils were stained with 0.1% aniline blue and pressed to desired thickness. Stigma and style were photographed under an upright fluorescence microscope (Zeiss AxioScope A1; Zeiss, Jena, Germany), and the ovule under a confocal laser scanning microscope (Zeiss LSM710; Zeiss, Jena, Germany) equipped with a Zeiss Axio Cam HRC camera (Zeiss, Jena, Germany).

### Transcriptome Sequencing

Thirty samples (Table 1) were processed for extraction of total RNA, purification of mRNA, and quantification as reported earlier (Jue et al., 2019; Lee et al., 2019). The quantified mRNA samples were then used to construct libraries. For this purpose, the mRNA was enriched with magnetic beads [together with Oligo (dT)], and it was randomly interrupted by adding fragmentation buffer, first strand cDNA was synthesized. The purified double-stranded cDNA was repaired, A-tailed, and connected to the sequencing adapter, and then AMPure XP beads were used for fragment size selection. Finally, the cDNA library is obtained by PCR enrichment. The quality of the libraries was determined by quantitative PCR, the libraries were pooled for each sampled tissue (pollination combination), and sequencing was performed on an Illumina platform.

**TABLE 1:** Details of longan samples used for transcriptome sequencing.

	Time after pollination (hours)			
Pollination combination	4	8	12	24
Yiduo ♀ × Shixia ♂(YS)	YS4	YS8	YS12	YS24
Shixia ♀ × Yiduo ♂(SY)	SY4	SY8	SY12	SY24
CK1	Yiduo sepal-pistil before pollination			
CK2	Shixia sepal-pistil before pollination			

### Bioinformatics Analyses of RNA-seq

Raw data were filtered to obtain clean data by removing reads containing connectors and low-quality reads in FastQC^2^. GC content distribution check was executed. The transcriptomic data were aligned with the longan reference genome (Lin et al., 2017) using HISAT2 (Kim et al., 2019). Comparison efficiency (percentage of mapped reads in clean reads) was computed and expressed as table and visualized in Integrative Genomics Viewer (Robinson et al., 2011).

Gene expression was quantified as fragments per kilobase of transcript per million fragments mapped (FPKM) and overall distribution of gene expression was expressed as a graph. Pearson’s correlation coefficient (PCC) and principal component analysis (PCA) were computed for the expression data between replicates of the treatments in prcomp^3^. Gene (read) count was calculated in order to obtain false discovery rate (FDR). After this, differentially expressed genes (DEGs) were screened using a criterion, i.e., log2 fold change ≥ 2 and FDR < 0.01. DEG-related analyses were performed in DESq2 (Love et al., 2014).

The DEGs were functionally annotated in different databases, i.e., KEGG (Kanehisa et al., 2004), gene ontology (GO) (Ashburner et al., 2000), clusters of orthologous groups (COG) (Tatusov et al., 2000), PfAM, Swissprot (Apweiler et al., 2004), egNOG (Huerta-Cepas et al., 2016), NR (Deng et al., 2006), and KOG (Koonin et al., 2004) using BLAST (Altschul et al., 1997). We then performed enrichment of DEGs in KEGG pathways in R/clusterProfiler (Version 3.10.1).

### RT-qPCR Analysis

To validate the RNA sequencing results, we selected 19 genes of interest (involved in JA metabolism) and performed RT-qPCR analyses. Primers were designed using Primer3Plus (Untergasser et al., 2007) (Table 2). qPCR setup and reaction conditions were as reported earlier (Luo et al., 2021). Relative gene expression was computed using the *GAPDH* gene as an internal control (Luo et al., 2021).

**TABLE 2:** List of primers used for real-time quantitative polymerase chain reaction (RT-qPCR) analyses of the selected genes.

Gene ID	Gene name	Forward primer sequence	Reverse primer sequence
*Dlo_022603.1.gene*	*PLA1*	ATCGTGGTCGTTGTCG	GAACTACAGAGGCGTGAG
*Dlo_020248.1.gene*	*PLA1*	ACGTTTGAGAGAA	AGAAATCACCCAGC
*Dlo_014311.1.gene*	*PLA1*	AGCTCCCACTACCT	TCAACTTCTGCCCCAC
*Dlo_031198.1.gene*	*LOX*	TTAGGCTATGGCGAGG	TAAGACGAGCA
*Dlo_006067.1.gene*	*LOX*	CCATGGTCAACCTCCTC	TCCACCTTTCATGTGCTC
*Dlo_012185.1.gene*	*LOX*	ACTCCGGTTAACAGC	TTACACACTACATTTC
*dlo_037902.1.gene*	*LOX*	GGTAGCAGGATCAATA	TGACCAAATGCAC
*Dlo_012184.1.gene*	*LOX13*	GAAGACATTTGAGA	ATTTAGGCCTAGTAT
*Dlo_001507.1.gene*	*LOX14*	TGTAGTGGGGTGGTAT	ATGTGGCATGAGGGG
*Dlo_015582.1.gene*	*AOS*	GATCGTGTTACATCA	TCTCAGCCTCAAAGT
*Dlo_011584.1.gene*	*AOC*	TGATTGGTGAAGCTCAA	TCATTGCACTGGC
*Dlo_024807.1.gene*	*OPR11*	CATCTGATGGCACA	TTACGAAGATGTGGG
*Dlo_010077.1.gene*	*OPR11*	ATTAGGGGCAATC	CCAACTCTTCGAAGTG
*Dlo_022217.1.gene*	*OPR11*	GTTGCAAGTTCCATACT	CAGCATAGCCAAGTCG
*Dlo_001987.1.gene*	*JMT*	ATCCTAAGGGATGC	AGGATATTTCGCTG
*Dlo_012262.1.gene*	*JAR1*	GCCAGTCATTCAAGCA	GCAGGTTCAAGCTTA
*Dlo_003095.1.gene*	*CYP94A2*	TCCACCCTATGCTCTCA	AGCCTCAATGGCAA
*Dlo_013441.1.gene*	*CYP94C1*	AGGCAGATATTCATAG	CTCAGAGCAGATAG
*Dlo_032545.2.gene*	*CYP94C1*	GTCCACATACTTCACG	AGAGAACAGATGAT
*GAPDH*		AACGTTGCCTGATTTT	GTACTTTCTTTCATACT

### Statistical Analyses

For biochemical (endogenous levels of the hormones) and phenotypic traits (time required by a tube to reach the end of style, length of style, and growth rate of pollen tube), means were compared in Microsoft Excel 2019 by one-way ANOVA. Difference at *p* < 0.05 was considered statistically significant. Standard deviation was also computed for each trait.

## Results

### Pollen Germination and Pollen Tube Growth

**FIGURE 2:**
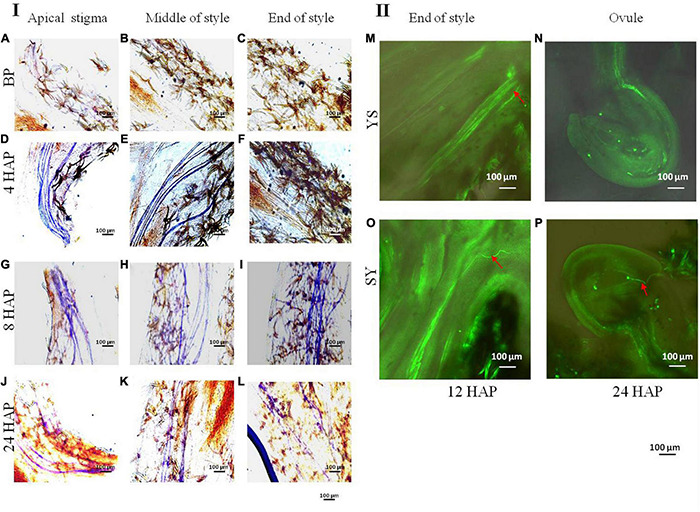
Observation of dynamic growth process of pollen tube. **(I)** Observation of dynamic growth process of pollen tube before and after pollination with Shixia pollen by *in situ* germination under an upright fluorescence microscope. **(A)** Stigma before pollination. **(B)** Middle of style before pollination. **(C)** End of style before pollination. **(D)** Stigma 4 h after pollination. **(E)** Middle part of style 4 h after pollination. **(F)** End part of style 4 h after pollination. **(G)** Stigma 8 h after pollination. **(H)** Middle part of style 8 h after pollination. **(I)** End part of style 8 h after pollination. **(J)** Stigma 8 h after pollination. **(K)** Middle part of style 8 h after pollination. **(L)** End of style 24 h after pollination. The pollen tube is blue after being stained with ultraviolet light. **(II)** Observation of the dynamic growth process of pollen tube by *in situ* germination method under a confocal laser scanning microscope in reciprocal crosses of Yiduo and Shixia. **(M)** Yiduo × Shixia 12 h after pollination. **(N)** Yiduo × Shixia 24 h after pollination. **(O)** Shixia × Yiduo 12 h after pollination. **(P)** Shixia × Yiduo 24 h after pollination. The red arrow points to the pollen tube.

The early datasets from our laboratory showed that in YS the fruit setting rate % is zero (Figure 1). To extend our observations, we explored some possibilities to which the UCI could be associated. First, we tested if the pollen of Shixia is viable/cannot germinate/sterile. To this regard, our microscopic observation showed that the pollens of both Yiduo and Shixia can germinate (Supplementary Figure 1). Second, we tested if the stigma of Yiduo is receptive or not. We found that the Yiduo stigma is receptive (Supplementary Figure 1), suggesting that the UCI is probably not due to defective stigma in Yiduo but another reason. Third, we tested if the pollen tube of Shixia can/cannot grow on the style of Yiduo. For this, we observed the *in situ* growth process of the pollen tube after pollinations in YS (Figure 2I). As expected, there was no growth of pollen tube prior to pollination (Figures 2A–C). In YS, the pollen tube elongated to the middle of the style 4 HAP (Figures 2D–F) and reached the end of the style 8 HAP (Figures 2G–I). However, 24 HAP, pollen tube signal became weaker (Figures 2J–L). This indicates that the pollen tube of Shixia can grow on the style of Yiduo. Finally, we observed that the pollen tube of Yiduo could enter into the ovule of Shixia, but in the case of Shixia pollen tube, it could not reach the ovule of Yiduo (Figure 2II). Altogether, the microscopic observations showed that the pollens of both Shixia and Yiduo can germinate, the stigmas of both cultivars are receptive, and the pollen tubes of both can growth into the style of the other. However, the final observation that Shixia pollen tube failed to reach Yiduo ovule is suspected to be the cause of the UCI (Figures 2N,P).

**FIGURE 3:**
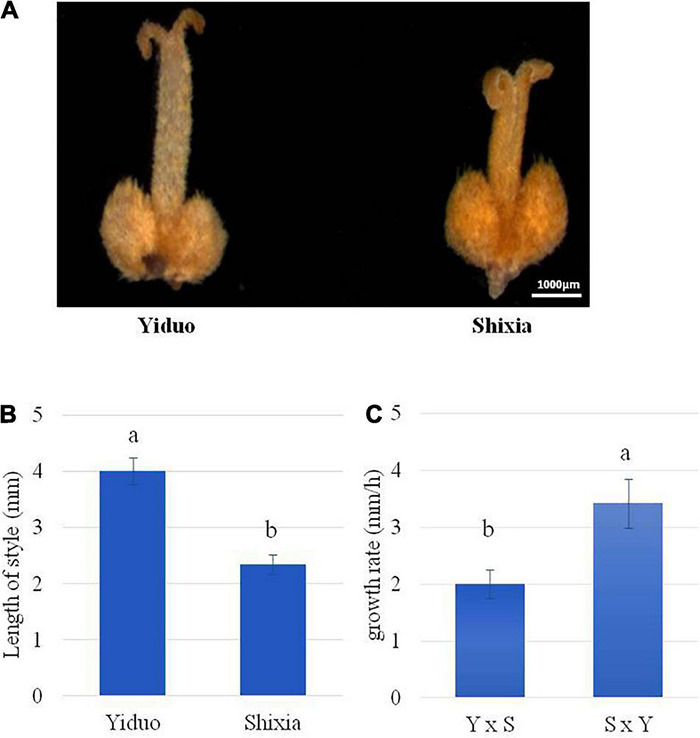
Comparison of the pistil and pollen tube of longan with a stereomicroscope. **(A)** Observation of the pistil of Yiduo and Shixia with a stereomicroscope. **(B)** Length of the style in mm and **(C)** comparison of pollen tube growth rate in the two reciprocal crosses. Y, Yiduo; S, Shixia. Different letters show that the means differ significantly (*p* < 0.05). Error bars represent the standard deviation.

To further understand the possible cause of the failure of Shixia pollen tube to reach Yiduo ovule, we measured the lengths of styles of Yiduo and Shixia. We observed that the lengths of the styles of Yiduo and Shixia are different, i.e., the length of Yiduo style is almost double of that of Shixia style (Figures 3A,B). This suggests that the failure of Shixia pollen tube to reach Yiduo ovule (Figure 2II) can be linked to the length of Yiduo style (Figures 3A,B). Besides, we also measured pollen tube growth rate in the two reciprocal pollination combinations. We found that the growth rate of the pollen tubes was significantly higher in SY than in YS (Figure 3C). Altogether, our observations propose that the observed IUC in YS is caused by the failure of the pollen tube of Shixia to enter the ovule of Yiduo, and this is probably due to the slow growth rate of the pollen tube of Shixia or the length of the style of Yiduo, or a combination of both phenomena.

### Transcriptome Sequencing

Our phenotypic and microscopic observations indicated chances of involvement of pollen tube growth and failure of pollen to reach the ovule in the case of YS pollination combination. Therefore, we further explored the transcriptomic signatures of both pollination combinations 4, 8, 12, and 24 HAP and compared them with the respective non-pollinated Yiduo (CK1) and Shixia (CK2) sepal-pistil.

**FIGURE 4:**
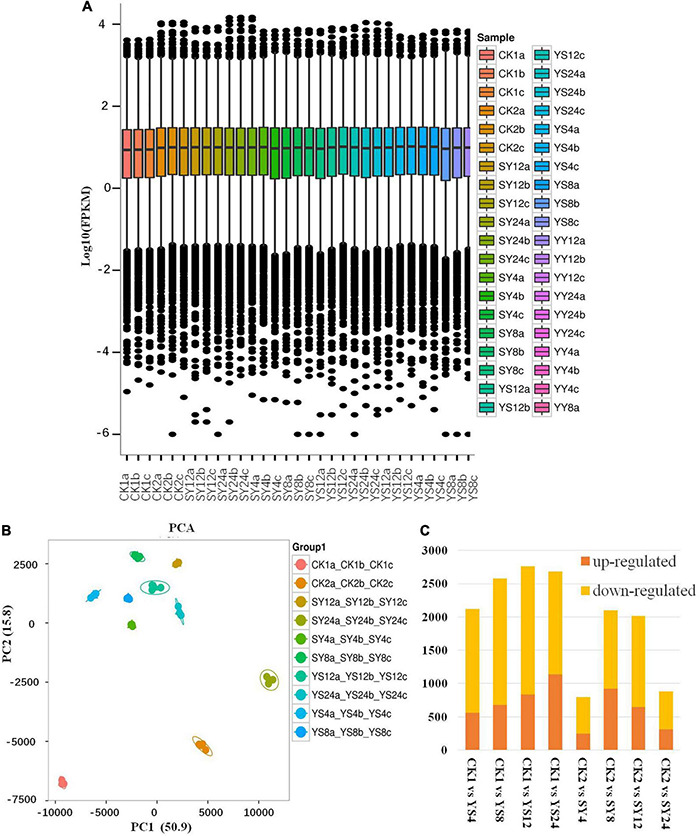
Transcriptional data analysis of Yiduo and Shixia. **(A)** Overall distribution of gene expression. **(B)** Principal component analysis of the gene expression data among the replicates of the treatments. **(C)** Summary of the differentially expressed genes in longan sepal pistils. Y, Yiduo; S, Shixia; CK1, Yiduo sepal-pistil before pollination, and CK2, Shixia sepal-pistil before pollination. The numbers with treatments represent time after pollination.

Transcriptome sequencing of the 30 libraries (Table 1) resulted in 271.9 GB clean data; on average, we got 5.71 Gb per sample with a Q30 base percentage of >93.7%. Comparison efficiency with the reference genome was 89.11 to 94%, and GC content was higher than 44%. On average, >90% of the reads could be mapped to the reference genome (Supplementary Table 1). Overall gene expression was lower in CK than in the other treatments (Figure 4A). The PCA showed that all biological replicates clustered together. In addition, all the treatments were grouped close to each other except for CK1, CK2, and SY24 (Figure 4B).

### Differential Gene Expression

Screening conditions for the DEGs were fold change ≥ 2 and FDR < 0.01. These conditions resulted in the identification of 7,251 DEGs in the studied longan pollination combinations. Overall, we observed that the number of downregulated DEGs was higher than that of upregulated DEGs in all the pollination combinations compared to their respective controls (Figure 4C). Relatively higher number of genes were differentially regulated between CK1 and YS time points indicating large scale transcriptomic regulation in YS as compared to SY in all time points as compared to their respective controls.

The KEGG pathway enrichment analysis showed that the DEGs were enriched in plant-hormone signal transduction pathway (93 DEGs), protein processing and endoplasmic reticulum (70 DEGs), and starch and sucrose metabolism (81 DEGs). Other than these, we also found enrichment of DEGs in phenylpropanoid biosynthesis pathway (66 DEGs). All these pathways were common in all the treatment comparisons (Supplementary Figure 2 and Supplementary Table 2).

**FIGURE 5:**
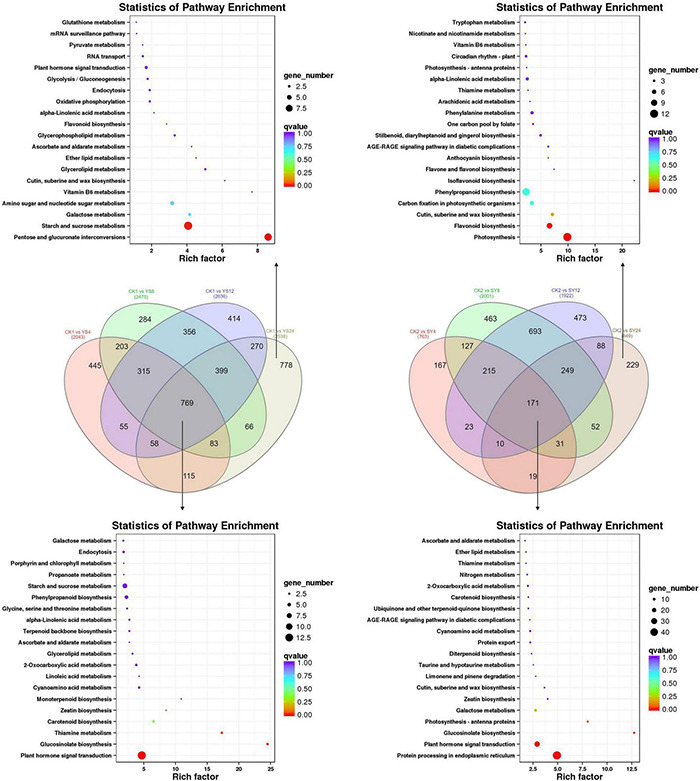
Differentially expressed genes in pollination combinations YS and SY. Venn diagrams show the common and specific DEGs in each pollination combination. KEGG enrichment scatter plots indicate the pathways in which common and specific DEGs were enriched. Y, Yiduo; S, Shixia; CK1, Yiduo sepal-pistil before pollination; CK2, Shixia sepal-pistil before pollination. The numbers with treatments represent time after pollination.

First of all, we compared the transcriptome sequencing results of both SY and YS (4, 8, 12, and 24 HAP) with their respective controls to find out DEGs associated with pollination. There were 769 DEGs common in all the time points in YS compared to CK1. Contrastingly, there were only 171 DEGs common in SY compared to CK2 (Figure 5 and Supplementary Table 3). These common DEGs could be related to overall development of the studied tissues in each of the pollination combinations. We found that 778 and 229 genes were specifically regulated in CK1 vs. SY24 and CK2 vs. YS24, respectively. This is the time point when we expect that the pollen will reach the ovule in the case of SY. The upregulated genes included ABC transporter G family members, ankyrin repeat-containing protein, *bHLH131*, *bHLH25*, and *WRKY33* TFs in SY24 compared to CK2. Interestingly, we also found the upregulation of an anther-specific protein, *LAT52*, in SY24 compared to CK2. This gene has been characterized for its roles in pollen development; however, its specific expression in SY24 might indicate its role in pollen tube development (Sheoran et al., 2007). Other than this, there were two genes, i.e., an ABC transporter and a later embryogenesis abundant protein (*Dimocarpus_longan_newGene_11049*), that were highly expressed in SY24 compared to CK2.

#### Differential Regulation of Plant-Hormone Signal Transduction Pathway

**FIGURE 6:**
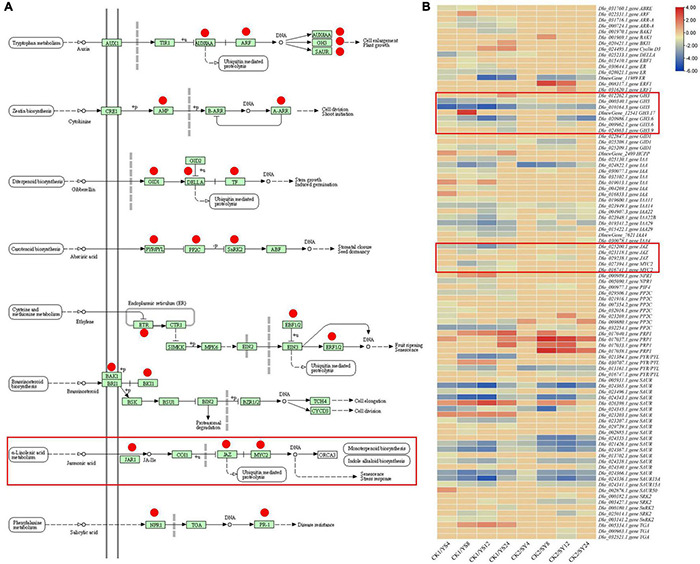
Comparison of hormone signal transduction pathway in the two longan pollination combinations. **(A)** Regulation of plant-hormone signal transduction pathway in two longan pollination combinations, i.e., YS and SY, compared to their respective controls. Y, Yiduo; S, Shixia; CK1, Yiduo sepal-pistil before pollination; CK2, Shixia sepal-pistil before pollination. The numbers with treatments represent the time (hour) after pollination. The red circles in the left panel represent the genes that were differentially regulated. Red rectangles represent the genes related to Jasmonic acid. **(B)** The right panel shows the heatmap of log2 fold change values of the DEGs between YS (4, 8, 12, and 24 HAP) vs. CK1 and SY (4, 8, 12, and 24 HAP) vs. CK2.

All the treatment comparisons showed KEGG pathway enrichment of plant-hormone signal transduction, suggesting an important role of this pathway in pollination. Therefore, we focused on the DEGs enriched in this pathway. Ninety-three DEGs were enriched in the plant-hormone signal transduction pathway (Supplementary Table 4; see DEGs highlighted in dark orange color; Figure 6). We observed that almost all the phytohormone signaling-related pathways were differentially regulated in at least one time point in either of the pollination combination. Interestingly, we found the specific regulation of 11 DEGs only in SY (DEGs with * in the Figure 6). These included BAK1, two ERF1s, GID1, IAA4, JAZ, PRP1, SAUR, SRK2, and two TGAs. On the contrary, a larger number of DEGs (42) were specifically expressed in CK1 vs. YS (4, 8, 12, and 24 HAP). These observations indicate that phytohormone signaling has specific roles in UCI in the pollination combination YS.

#### Differential Expression of Jasmonic Acid Metabolism

**FIGURE 7:**
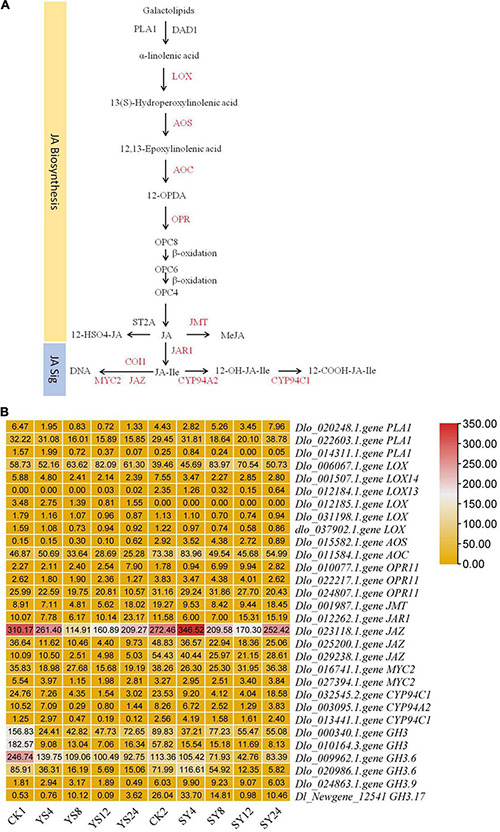
Differential regulation of jasmonic acid metabolism and signaling in the YS and SY pollination combinations compared to their respective controls. **(A)** The pathway of Jasmonic acid metabolism and signaling. The red colored text represents the differentially regulated genes in the pathway. DAD1 (DEFECTIVE IN ANTHER DEHISCENCE 1), LOX (lipoxygenase), AOS (allene oxide synthase), AOC (allene oxide cyclase), OPR (OPDA Reductase), JMT (JA methyl transferase), ST2A (sulfotransferase 2A), JAR1 (Auxin-responsive GH3 family protein; jasmonic acid-amino synthetase), COI1 (coronatine-insensitive protein 1), JAZ (Jasmonate ZIM domain-containing protein), MYC2 (transcription factor MYC2), CYP94A2 (jasmonoyl-isoleucine 12-hydroxylase), and CYP94C1. Y, Yiduo; S, Shixia; CK1, Yiduo sepal-pistil before pollination; CK2, Shixia sepal-pistil before pollination. The numbers with treatments represent time after pollination. **(B)** The heatmap shows the FPKM values of differentially regulated genes.

As reviewed in the section “Introduction,” JA levels have been related to limited pollen germination in Arabidopsis (Ju et al., 2016). Therefore, we explore JA metabolism to see the differential transcriptome signature in both pollination combinations. The conversion of galactolipids into α-linoleic acid by PLA1 and DAD1 initiates JA metabolism in plants (Ishiguro et al., 2001). Although we did not observe the differential expression of DAD1 gene between the studied samples, we observed that two PLA1 genes (*Dlo_020248.1.gene* and *Dlo_022603.1.gene*) had relatively lower expression in the YS series compared to the CK1 and SY time points. Particularly, their expression was nearly half in YS compared to SY (Figure 7). In addition, we observed that six transcripts annotated as LOXs were differentially regulated in the studied tissues. Of these, one LOX (*Dlo_012185.1.gene*) was specific to YS, and another was specific to SY (*Dlo_012184.1.gene* LOX13), while the others were common in both pollination combinations. The expression trend of the LOX genes was similar in both YS and SY pollination time series. However, two LOXs (*Dlo_006067.1.gene* and *Dlo_012185.1.gene*) had globally higher FPKM values in YS than in SY, suggesting that 24 HAP, the level of JA is higher in YS than in SY (Figure 7). AOS and AOC control the conversion of 13(s) hydroperoxylinoleic acid to 12-13-epoxylinoleic acid to 12-oxophytodienoate (12-OPDA), respectively. 12-OPDA is then reduced to OPC8 (3-oxo-2-(2-pentenyl)-cyclopentane-1-octanoic acid). This reaction is controlled by OPR genes (OPDA reductase) (Wasternack and Hause, 2013) and finally leads to JA biosynthesis. In this study, we identified one AOS gene with a conspicuous different expression pattern between YS and SY. We observed that the transcript annotated as AOS (*Dlo_015582.1.gene*) had globally higher expression in YS than in CK1. However, in SY, its expression was globally lower than in CK2. The transcriptome analysis also showed three transcripts annotated as OPR11 (*Dlo_010077.1.gene, Dlo_022217.1.gene*, and *Dlo_024807.1.gene*) that were differentially expressed in the studied tissues. Only *Dlo_010077.1.gene* showed an obvious difference between YS and SY. The comparison between YS24 and SY24 indicated a ∼three-fold difference (YS24 had higher expression), suggesting high conversion of 12-OPDA to OPC8 in YS. Collectively, higher expression of JA biosynthesis genes in YS than in SY was observed, which implies high JA level in YS tissues. JA is converted into MeJA by JA carboxyl methyltransferase (JMT) (Seo et al., 2001). The transcriptome sequencing showed that a JMT gene (*Dlo_001987.1.gene*) had reduced expression in YS4, YS8, and YS12 compared to CK1, but that its expression significantly increased in YS24 compared to CK1. In the case of SY, its expression decreased in all the four time points compared to CK2; however, the expression in SY24 was relatively higher 4, 8, and 12 HAP. The expression in both combinations was quite similar 24 HAP, proposing a similar concentration between the two combinations 24 HAP. Overall, the comparative expression analysis directs that the levels of JA might be higher in the YS time points than those in the SY pollination combination (the JA content in the studied comparison is presented below in section “Endogenous Jasmonic Acid Levels in Pistils of Yiduo and Shixia”) (Figures 6, 7).

#### Differential Expression of Jasmonic Acid Signaling

Since JA signaling has been shown to be involved in the regulation of genes associated with pollen germination, pollen tube growth, and cross incompatibility (Muradoğlu et al., 2010; Guan et al., 2013; Chaturvedi et al., 2016), we explored the changes in the expression of genes that were involved in JA biosynthesis and signaling. JA signaling is a part of the plant-hormone signal transduction pathway (Yang et al., 2019). JA-Ile is formed by jasmonoyl-isoleucine synthetase 1 (JAR1), which is a member of the GH3 gene family (Staswick and Tiryaki, 2004). This is the major step in JA perception, since JA-Ile is the most biologically active JA compound (Fonseca et al., 2009). The expression of JAR1 was lower in YS4 and YS8 than in CK1, but it was increased in YS12 and YS24; in particular, the expression was significantly higher 24 HAP, suggesting higher conversion of JA into JA-Ile at this time point. A similar expression pattern was noted in the four time points of the SY pollination combination. The main difference between both pollination combinations was that YS24 had higher FPKM values than SY24, proposing that YS24 might have higher JA-Ile concentration than SY24. JA-Ile signal is perceived by coronatine-insensitive protein 1 (COI), which triggers the repression of JAZ. In both pollination combinations, the expression of JAZs decreased as compared to their respective controls, indicating that JA-Ile triggered the repression of JAZs, although we did not note the differential regulation of COI. JAZ repression releases downstream transcription factors (TFs), e.g., MYC2s. We noted that two MYC2s were differentially regulated between the two pollination combinations. Both MYC2 genes (Dlo_016741.1.gene and Dlo_027394.1.gene) were downregulated in YS as compared to CK1, whereas only downregulated in SY4 and SY8 but upregulated SY12 and SY24 as compared to CK2. This observation proposes that in the YS pollination combination, the JA-responsive activation of MYC2 is relatively lower than that of SY (in particular 12 and 24 HAP). There are multiple genes present downstream of MYC2s that regulated JA-triggered responses in plant tissues (Ruan et al., 2019) (Figures 6, 7). Overall, considering the regulation of JA metabolism and signaling-related genes in the two pollination combinations, it can be proposed that JA biosynthesis and signaling have an important role in UCI in YS.

The expression pattern of 19 genes involved in JA metabolism was also confirmed by RT-qPCR analysis (Supplementary Figure 3), which was consistent with the FPKM values of these genes. Theses expression profiles signify both the reliability of the RNA sequencing and the role of JA metabolism.

#### Differential Regulation of Phenylpropanoid Pathway, Cell Wall, and Pollen Tube-Related Genes

**FIGURE 8:**
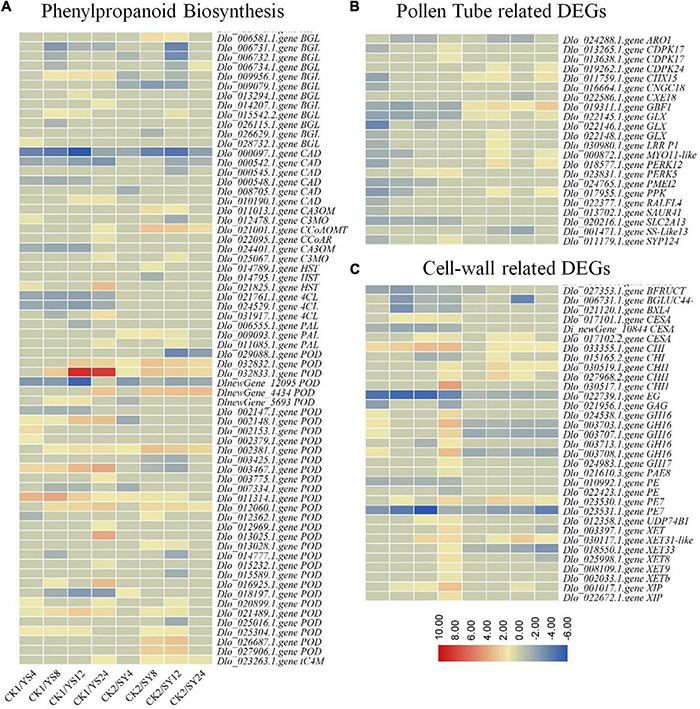
**(A)** Differential regulation of phenylpropanoid biosynthesis pathway, **(B)** pollen tube-related, and **(C)** cell wall-related genes in the CK1 vs. YS series and CK2 vs. SY series. The heatmaps show log2 fold change values of the DEGs. Y, Yiduo; S, Shixia; CK1, Yiduo sepal-pistil before pollination; CK2, Shixia sepal-pistil before pollination. The numbers with treatments represent time after pollination.

The KEGG pathway analysis indicated that 66 DEGs were enriched in the phenylpropanoid pathway. The DEGs included multiple genes that control the main steps of the phenylpropanoid biosynthesis pathway. Three β-glucosidase (BGLs), a caffeic acid (3-O-methyltransferase, CA3OM), two CADs, two shikimate O-hydroxycinnamoyltransferase (HSTs), a phenylalanine ammonia-lyase (PAL), and seven peroxidases (PODs) were specific to SY 8 and 12 HAP. Similarly, 21 DEGs were specific to YS. We observed that of the three 4CLs, the expression of two transcripts (*Dlo_021761.1.gene* and *Dlo_024529.1.gene*) was significantly decreased in all the YS time points, whereas the expression of the third transcript increased significantly in YS24 compared to CK1 and YS4-12. Contrastingly, the expression of the three 4CL transcripts decreased at SY4 and SY8 but then returned to nearly the same as of CK2 (but remained lower than CK2). One CA3OM transcript showed an expression pattern similar to that of 4CLs. The biosynthesis of coumarine might be similar in both pollination combinations because of increase in the expression of BGLs. BGLs are known for their roles in the biosynthesis of coumarins (Schaeffer et al., 1960). How coumarin biosynthesis may/may not affect pollen tube elongation is less known (Adhikari et al., 2020). However, current observations propose that coumarins have a limited role in pollen tube elongation. We also found the differential expression of PALs, where two of the three PAL transcripts showed reduced expression compared to the controls, while the third transcript showed increased expression. This similar expression trend between both pollination combinations might suggest that PAL might not be a key stage for the control of the biosynthesis of lignin in longan. This is understandable, since PAL controls the first stage of phenylpropanoid biosynthesis, i.e., phenylalanine conversion into cinnamic acid (Zhang and Liu, 2015) (Supplementary Table 4 and Figure 8). Our transcriptome sequencing also showed the differential expression of a transcript annotated as myo-inositol transporter 13 (INT13). Its expression decreased in both YS and SY series compared to CK1 and CK2, respectively; however, its expression in YS24 was lower than in SY24.

Since, pollen tube is a fast-growing cell and requires higher amounts of cell wall deposition, it is also essential to check the expression of cell wall-related genes. We selected all the DEGs (33) containing the term “cell-wall” based on GO annotation and checked their differential regulation in SY and YS. Of the 33 cell wall-related DEGs, 15 were specific to YS. We found that the expression of a cellulose synthase A (CESA, *Di_newGene_10844*), galacturan 1,4-alpha-galacturonidase (GAG, *Dlo_021956.1.gene*), and a pectinesterase (PE, *Dlo_010992.1.gene*) was reduced in SY (4, 8, 12, and 24 HAP) compared to CK1. These expression changes may suggest that the deposition of cell wall in the growing pollen tube might be lower in the case of YS. However, pollen tube growth in both SY and YS, at least until 12 HAP, could be due to the higher expression of a chitinase (CHI, *Dlo_030519.1.gene*) and a CESA (*Dlo_017102.2.gene*). Particularly, we found that *Dimocarpus_longan_newGene_10844* (CESA) expressions were reduced compared to CK1 in all the four time points. Furthermore, its expression remained almost unchanged in SY4-8, slightly decreased in SY12, and then increased in SY24 (although in this stage, the expression was still lower than in CK2). In YS24, its expression was lower than that in SY24. Other than CESAs, we found that the expression of most of the xyloglucan:xyloglucosyl transferases (XETs) was higher in YS24 than in CK1 and at all the SY time points. Similarly, we noted the differential expression of xylanase inhibitors (XIPs, *Dlo_001017.1.gene* and *Dlo_022672.1.gene*), pectin methylesterase (PME, also known as PE, *Dlo_023530.1.gene*), and PME-inhibitor (PMEI, *Dlo_024765.1.gene*).

We also searched for the term “pollen tube” to screen the DEGs associated with pollen tube and found 22 genes that were differentially expressed in CK1 vs. YS and CK2 vs. SY at different time points after pollination. We noted that a cation/H(+) antiporter 15 (CHX15, *Dlo_011759.1.gene*) showed higher expression in SY24 than in CK2, whereas its expression first reduced 4,8, and 12HAP in YS but then slightly increased (although not differentially expressed) in YS24 compared to CK1. Regarding our search against the term “pollen tube,” we found the differential expression of many Ca^2+^ signaling-related genes. Particularly, we found the differential expression of three calcium-dependent protein kinase (CPK) genes (CPK24 and two CPK17), a cyclic nucleotide-gated channel (CNGC18), and a syntaxin-124 (SYP124) gene. CPK17s were highly expressed in the YS time points (particularly, their expression increased in YS24 compared to CK1), whereas their expression was only fractional in the SY time series. CNGC18 (*Dlo_016664.1.gene*) showed higher gene expression in YS24 than in SY24. The transcriptome data also revealed the higher expression of SYP124 in YS than in SY. This expression trend is similar to that of other genes affected by Ca^2+^ signaling. Other than these, we noted the differential expression of a protein RALF-like 4 (RLAFL4, *Dlo_022377.1.gene*), a pollen-specific leucine-rich repeat extensin-like protein 1, STRICTOSIDINE SYNTHASE-LIKE 13 (SS-like 13, *Dlo_001471.1.gene*), an armadillo repeat only 1 (ARO1, *Dlo_024288.1.gene*), and two proline-rich receptor-like protein kinases (PERK12, *Dlo_018577.1.gene* and PERK5, *Dlo_023831.1.gene*). The RALF protein is known for male sterility (Zou et al., 2017), whereas PERKs are known for pollen tube growth (Borassi et al., 2021). RLAFL4, SS-like 13, and PERKS should be given due consideration in future studies exploring UCI in longan based on the expression patterns observed in our study.

#### Differential Regulation of Starch and Sucrose Biosynthesis Pathway

Earlier reports have presented that sucrose concentration affects significantly pollen tube growth (Fragallah et al., 2019). Considering that we observed changes in cell wall biosynthesis, loosening, and remodeling-related genes (see section “Differential Regulation of Phenylpropanoid Pathway, Cell Wall, and Pollen Tube-Related Genes”), in addition, we checked the differential expression of genes (50 DEGs) that were significantly enriched in the starch and sucrose biosynthesis pathway. Interestingly, we found that three of the four DEGs annotated as sucrose synthases (SuSy, *Dimocarpus_longan_newGene_4925*, *Dlo_005657.1.gene*, and *Dlo_013696.1.gene*) were downregulated in YS compared to CK1. On the contrary, two of the four transcripts showed upregulation in SY, *Dimocarpus_longan_newGene_4925* and *Dimocarpus_longan_newGene_11275*), whereas one gene (*Dlo_005657.1.gene*) had stable expression in the SY series compared to CK2. Two sucrose phosphate synthases (SPSs, *Dlo_015436.1.gene* and *Dlo_022163.1.gene*) also showed decreased expression in YS compared to CK1, in contrast to the upregulation of the second transcript in SY compared to CK2 (Supplementary Table 4). These observations are consistent with the observation that in YS, pollen tube growth rate was lower than in SY (Figure 3C).

The 1,4-alpha-glucan branching enzyme (GBE, *Dlo_028752.1.gene*) gene was upregulated in all the four time points of both pollination combinations compared to their respective CKs. Two hexokinases (HKs, *Dlo_000488.1.gene* and *Dlo_027870.1.gene*) were downregulated in the YS series compared to CK1, whereas in SY, the first transcript was upregulated. Another enzyme fructokinase (FRK, *Dlo_022066.1.gene* and *Dlo_033973.1.gene*) that controls the same step as of HK i.e., conversion of D-fructose to D-fructose-6P (Jiang et al., 2003; Kawai et al., 2005), was downregulated in YS as compared to CK1 and upregulated in SY as compared to CK2. However, β-fructofuranosidase (BFRUCTF) showed decreased expression in both pollination combinations, indicating depletion of sucrose (Schwebel-Dugué et al., 1994). We also noted the differential expression of multiple genes that take part in D-glucose biosynthesis in the starch and sucrose biosynthesis pathway. The breakdown of cellulose to cellodextin by endoglucanase (EG, *Dlo_022739.1.gene* and *Dlo_028821.1.gene*) (Opassiri et al., 2006) and then to cellobiose by EG and beta-glucosidases (BGLs) (Yoshida and Komae, 2006) leads to D-glucose biosynthesis. The expression of EGs decreased in both YS and SY compared to CK1 and CK2, whereas the expression of BGLs was variable in both cases (Supplementary Table 4).

Finally, there were changes in the expression of genes related to starch metabolism. Glucose-1-phosphate adenylyltransferase (G1P), which converts α-D-glucose-1P to ADP-glucose (Ghosh et al., 1992), showed a similar expression pattern in both pollination combinations. Similarly, the gene that controls the biosynthesis of starch/glycogen from amylose i.e., 1,4-alpha-glucan branching enzyme (Baecker et al., 1986) was upregulated in all YS series as compared to CK1. However, this gene was downregulated in SY series (except for SY24). These expression changes indicate that the starch biosynthesis is higher in all YS time points but not in SY series (except for SY24) as compared to CK1 and CK2. Our transcriptome data showed that the expression of α-amylase (AAs) increased in the YS series compared to CK1. On the contrary, only one AA (*Dlo_001166.1.gene*) was upregulated in the SY series. The expression of two of the three β-amylases (BAs) (*Dlo_026796.1.gene* and *Dlo_029595.1.gene*) was reduced in YS compared to CK1 in all the time points. On the contrary, the expression of the two above-mentioned BAs was reduced in SY4-12 but significantly increased in SY24 compared to CK2 and CK1 (Supplementary Table 4).

#### Differential Regulation of Protein Processing in the Endoplasmic Reticulum

Pollen tube growth needs delivery of a wide variety of proteins and requires intense streaming and trafficking (Scali et al., 2021). In this regard, it is essential for a growing cell to process proteins in the endoplasmic reticulum. The KEGG pathway enrichment analysis indicated that 70 DEGs were enriched in protein processing in the endoplasmic reticulum pathway in both pollination combinations. Of the 70 DEGs, 40 were specifically expressed in CK1 vs. YS. Among the DEGs, two (HSP70KDa 1/8, *Dlo_032043.1.gene* and molecular chaperon HtpG, *Dlo_033047.1.gene*) showed interesting expression profiles, i.e., they were downregulated in all the treatment comparisons except in CK2 vs. SY24. Hsp70KDas are known for their involvement in polar nuclei fusion during female gametophyte and sperm nuclear fusion with central cell polar nuclei at fertilization (Maruyama et al., 2010). Also, they are required for pollen development and pollen tube growth (Maruyama et al., 2014). On the other hand, the role of HtpGs in pollen tube development is not elaborated yet. Nevertheless, HtpGs have been implicated in heat stress endurance of pollen tubes (Chaturvedi et al., 2016) (Supplementary Table 4).

### Endogenous Jasmonic Acid Levels in Pistils of Yiduo and Shixia

**FIGURE 9:**
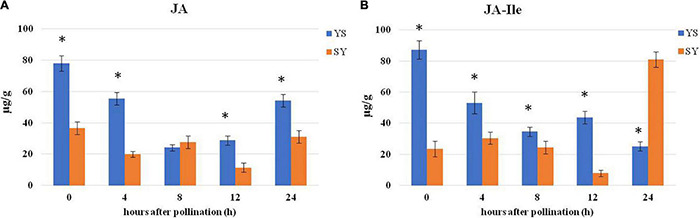
Comparison of hormone levels in pistils of SY and YS. **(A)** Jasmonic acid content. **(B)** JA-Ile content 0, 4, 8, 12, and 24 h after pollination. YS, Yiduo♂ × Shixia♀; SY, Shixia♀ × Yiduo♂. Asterisk on the bars show significant differences (*p* < 0.05).

Using ab SCIEX QTRAP 6500 LC-MS/MS technique, we measured the changes in JA and JA-Ile contents in the two pollination combinations, i.e., SY and YS, before and after 4, 8, 12, and 24 HAP (Figure 9). We observed that, overall, the JA levels were higher in YS than in SY (Figure 9A). A similar trend was noticed for the endogenous JA-Ile levels (Figure 9B). These observations propose that JA might be involved in UCI.

## Discussion

### Cell Wall-Related Genes That Are Associated With Changes in Pollen Tube Growth Are Differentially Expressed in Shixia♀ × Yiduo♂ and Yiduo♀ × Shixia♂

There are many requirements for successful fertilization in plants, i.e., pollen germination, receptivity of the stigma, pollen tube growth, and successful transmission of pollen (Krichevsky et al., 2007). Our observations that the pollens of both Shixia and Yiduo germinated normally, and that the stigma of the Yiduo was receptive (Supplementary Figure 1) show that these two pre-fertilization reproductive barriers are not different in the studied pollination combinations, SY and YS (Deng et al., 2017). The microscopic observation of the dynamic process of pollen tube growth indicated that when a Shixia pollen is used to fertilize a Yiduo stigma, signals of pollen tube growth weaken 24 HAP. This was further confirmed by observing the arrival of the pollen tube in the ovule 12 and 24 HAP. These results indicated that in the case of SY, the pollen tube reached the ovule within 24 HAP; however, in the case of YS, its growth rate was slower (Figure 2II). These microscopy-based observations propose that (1) stigmas of both S and Y are receptive; (2) in the case of SY, the pollen tube elongates and reaches the end of style in 24 h; (3) whereas in the case of YS, the pollen tube elongates into the style by 24 h but fails to reach the ovule, indicating that the lower pollen tube growth in YS than in SY is one of the reasons of UCI in YS. The notable transcriptome change that is relevant to this microscopic observation is the expression of 4CLs and CA3OM. 4CLs controlling the essential steps of the biosynthesis of secondary metabolites leading to lignin (Hamberger and Hahlbrock, 2004). Their expression, together with CA3OM, indicates that lignin or other downstream metabolites in YS24 must be decreased, whereas in SY it was almost normal. This is relatable to the known fact that a massive cell wall deposition is required for fast elongation of pollen tubes, and that we observed faint pollen tube signals in YS24 (Figure 2). Earlier studies explained that the deposition of lignin was affected by changes in the expression of 4CLs and CA3OM (Ma and Xu, 2008; Cao et al., 2020). Thus, these genes would be a suitable candidate for increasing lignin deposition in elongating pollen tubes.

The expression changes in CESAs suggests two things, i.e., first, cellulose synthesis in YS is affected similar to the lignin biosynthesis and, second, that YS24 may have lower cellulose deposition than SY24 (Nawaz et al., 2019). The second observation could not be generalized, since two other CESAs showed higher expression in YS24 than in CK1, and their expression pattern was similar in both pollination combinations. The observation that there were three CESA transcripts could be due to the fact that there are multiple CESA members that differ in function and are expressed differently (Nawaz et al., 2017a,^2019^). Furthermore, in Arabidopsis, the silencing of *CESA1* and *CESA*3 caused no pollen tube production (Persson et al., 2007). Only a further experiment might reveal if *Dimocarpus_longan_newGene_10844* or the other two also have a possible role in UCI in the YS pollination combination. Xyloglucan is a major hemicellulosic polysaccharide of a primary cell wall, which interacts with cellulose and controls cell wall expansion. Our observation that the expression of most of the XETs was higher in YS24 than in CK1 and all the SY time points is in line with earlier reports that XET activity loosens plant cell walls (Van Sandt et al., 2007; Nawaz et al., 2017b). It suggests, that in the YS pollination combination, the pollen tube could be under modification higher than that of the SY pollination combination. This proposition is further supported by the expression pattern observed for XIPs (*Dlo_001017.1.gene* and *Dlo_022672.1.gene*), as XIPs are known for degradation of xylans and the fact that in rice they are downregulated during pollen tube growth (Dai et al., 2007). In addition, it is known that the application (or increased expression) of PMEs causes a dramatic decrease in pollen tube germination/growth (Bosch et al., 2005). One PME (also known as PE, *Dlo_023530.1.gene*) showed higher expression in YS24 (8.58) than in SY24 (3.06). The expression pattern of one PME (*Dlo_023530.1.gene*) is consistent with the known role of PMEs in *Nicotiana tabacum* (Bosch et al., 2005). In accordance with this, the differential expression of PMEI (*Dlo_024765.1.gene*) in YS and SY compared to CK1 and CK2, respectively, indicates that in YS the PME level might be higher because of the increased expression of PME/PE as a result of the reduced expression of PMEI and vice versa in SY. We say this because PMEI inhibits PME from flower, siliques, and pollens (Wolf et al., 2003). Taken together, our transcriptome results show that genes associated with cell wall biosynthesis, modification, and lignin deposition express differentially between both YS and SY pollination combinations, and are one of the reasons for UCI in YS. These results lay down the foundations of specific future investigations targeting cell wall modification during YS pollen tube growth compared to that of SY.

### Differential Expression of Pollen Tube Growth-Related Genes Was Observed Between Shixia♀ × Yiduo♂ and Yiduo♀ × Shixia♂

Pollen tubes are fast-growing cells which require multiple factors. *In vitro* pollen tube growth studies have highlighted that Ca^2+^, K^+^, and pH dynamics are prime factors that accompany tip growth (Hepler et al., 2006; Scheible and McCubbin, 2019). Thus, ion transporters are prime targets to understand if there are differences in pollen tube growth rate, as observed in our study (Figures 2, 3). The observation that a CHX15 was upregulated in the SY series compared to CK2 and downregulated in the YS series compared to CK1 is relevant to recent findings that two CHXs (CHX21 and CHX23), when mutated, result in normal pollen germination but are defective in pollen tube guidance to the ovule (Lu et al., 2011). CHX15, CHX21, and CHX23 belong to same subfamily of cation exchangers [see UniProtKB - Q9SIT5^4^; (Chanroj et al., 2011)]; therefore, a similar role can be expected. This is an interesting observation and a good gene of interest for a detailed exploration of its role in longan UCI. Studies have elaborated that K^+^ is required to regulate turgor pressure in pollen tubes to burst during fertilization (Holdaway-Clarke and Hepler, 2003), and that K^+^ influx promotes pollen tube growth (Mouline et al., 2002). At a relatively higher Ca^2+^ concentration, the mutation of CPKs did not inhibit pollen tube growth in Arabidopsis, indicating that CPKs (CPK11 and CPK24) control pollen tube growth. Our observations that the expression of two CPK17s was higher in YS than in CK1 and lower in SY than in CK2 can be relevant to this known fact. Particularly, the higher CPK24 expression in YS24 than in SY24 is consistent with this report (Zhao et al., 2013). Ca^2+^ channels are core components in pollen tube tips that regulate Ca^2+^ gradients. Mutations in CNGC18 have been studied in Arabidopsis pollen tube growth and were found critically responsible for pollen tube guidance. The similar expression trend of the CNGC18 (Gao et al., 2016) indicates its lesser role in UCI in the YS longan pollination combination. Another protein whose localization is influenced by Ca^2+^ is syntaxin (Silva et al., 2010). In our data, the higher expression of SYP124 in YS than in SY is consistent with an earlier report as well as the expression pattern of CPKs (Silva et al., 2010). The expression of 1-phosphatidylinositol-4-phosphate 5-kinase is also consistent with the known function that homozygous mutants lacking *PIP5K4* transcript significantly affect pollen tube growth and polarity (Sousa et al., 2008). Finally, pollen tube growth has also been related with Arabidopsis inositol transporter 4 (INT4) (Schneider et al., 2006). The expression of INT13 is consistent with the expression in Arabidopsis that INT4 was detected in growing pollen tubes and plays a role in high rate of pollen rate growth (Schneider et al., 2006). Taken together, our observations from transcriptome sequencing and comparison indicate that many genes associated with pollen tube growth were differentially expressed between SY and YS and cannot be ignored particularly in relation to the microscopic observations made in our report.

### Starch and Sucrose Biosynthesis Pathway Is Differentially Regulated in the Yiduo♀ × Shixia♂ and Shixia♀ × Yiduo♂ Pollination Combinations

Fast-growing pollen tubes require different resources such as sugars, cell wall polymers, and energy. It has been established that sucrose content is positively correlated with pollen germination as well as pollen tube growth in *N. tabacum* (Parrotta et al., 2018) and *Cunnighamial lanceolata* L (Fragallah et al., 2019). Thus, regulation of the starch and glucose biosynthesis pathway can highlight potential genes that play a role in UCI in the YS longan pollination combination. The downregulation of SuSy and SPS in YS compared to SY is consistent with our observations on pollen tube growth rate (Figures 2, 3) and changes in the expression of genes related to pollen tube and cell wall (see above sections). Both the SuSy and SPS genes are required for sucrose metabolism (Winter and Huber, 2000), and their expression changes indicate that this process might be disturbed in the YS pollination combination compared to SY. The expression of HKs and FRKs indicate that in YS, the conversion of D-fructose to D-fructose-6P is reduced compared to the SY pollination combination. This statement is based on the known function of both enzymes (Jiang et al., 2003; Kawai et al., 2005). However, transcripts for a gene present upstream this step, i.e., BFRUCTF, showed decreased expression in both pollination combinations, indicating depletion of sucrose (Schwebel-Dugué et al., 1994), which shows that sucrose breakdown is similar in both pollination combinations. The breakdown of sucrose also results in D-glucose (Schwebel-Dugué et al., 1994). In this regard, we noted the differential expression of multiple genes that take part in D-glucose biosynthesis in the starch and sucrose biosynthesis pathway. The breakdown of cellulose to cellodextin by endoglucanase (EG, *Dlo_022739.1.gene* and *Dlo_028821.1.gene*) (Opassiri et al., 2006) and then to cellobiose by EG and beta-glucosidases (BGLs) (Yoshida and Komae, 2006) leads to D-glucose biosynthesis. The variable expressions of EGs and BGLs enable us to think that there are changes happening in cellulose and its conversion to D-glucose. Apart from sucrose and D-glucose metabolism-related transcriptome changes, the fact that multiple genes controlling starch metabolism, i.e., 1,4-aplha-glucan branching enzyme, AAs, and BAs were differentially regulated between YS and SY, indicating changes in starch concentration in the studied tissues. Most probably, we can understand that starch/glycogen biosynthesis in YS must be higher than in SY, and that its conversion to maltose and/or destrin is also higher in YS than in SY. However, these expression changes cannot be considered a direct cause of UCI in YS in comparison to SY.

### Jasmonic Acid Metabolism and Signaling Are Regulated Differently in the Yiduo♀ × Shixia♂ and Shixia♀ × Yiduo♂ Pollination Combinations

Since MeJA is known to affect pollen germination as well as pollen tube growth (Muradoğlu et al., 2010), we specifically explored the JA metabolism and signaling pathways. Muradoğlu, Yıldız and Balta (Muradoğlu et al., 2010) reported that MeJA led to a decrease in pollen germination as well as pollen tube growth in apricots. Another study reported that the application of 2.5 mM MeJA resulted in no pollen germination in *Pinus nigra* (Çetinbaş-Genç and Vardar, 2020). Our observations that both JA metabolism and signaling were differentially regulated in both the YS and SY pollination combinations are in accordance with these reports. Most importantly, in JA metabolism, the conversion of galactolipids into α-linoleic acid is controlled by PLA1 and DAD1 (Ishiguro et al., 2001). The expression changes in PLA1 indicate that the conversion of galactolipids into α-linoleic acid is higher in SY and relatively lower in YS. This was further confirmed by the expression trend of PLA1, as noted in the RT-qPCR analysis (Supplementary Figure 3). Next step that leads the biosynthesis of JA is the LOX pathway, i.e., AOS branch (Feussner and Wasternack, 2002). The expression patterns of genes that control important steps of the LOX pathway, i.e., LOXs, OPR11, and JMT in YS (particularly at YS24) compared to SY are consistent with observed changes in endogenous JA and JA-Ile levels based on the known functions of the respective genes (also see Supplementary Figure 3) (Ishiguro et al., 2001; Seo et al., 2001; Feussner and Wasternack, 2002; Wasternack and Hause, 2013). JA-Ile is formed by JAR1, which is a member of the GH3 gene family (Staswick and Tiryaki, 2004). This is a major step in JA perception, since JA-Ile is the most biologically active JA compound (Fonseca et al., 2009). Its higher expression in YS is consistent with the higher JA-Ile concentration (Figure 7 and Supplementary Figure 3). JA-Ile signal is perceived by COI, which triggers the repression of JAZ. The transcriptome comparisons showed that JA-Ile triggered the repression of JAZs in both YS and SY, although we did not note the differential regulation of COI. JAZ repression releases downstream transcription factors (TFs), e.g., MYC2s. The expression pattern of MYC2s indicates that in the YS pollination combination, the JA-responsive activation of MYC2 is relatively lower than that in SY (in particular 12 and 24 HAP) (Supplementary Figure 3). There are multiple genes present downstream of MYC2s that regulate JA-triggered responses in plant tissues (Ruan et al., 2019). These results are in accordance with the fact that the downregulation of these genes has been linked with the inhibition of endogenous JA synthesis in Arabidopsis during pollen germination (Ju et al., 2016). Taken together, the changes in the expressions of JA biosynthesis- and signaling-related genes indicate an important role of JA in UCI. However, further studies involving exogenous application of JA/MeJA or agents that can regulate its *in planta* biosynthesis will elaborate its role. Also, characterization of genes through gene silencing or other techniques would be useful to explain clearly how the expression of the individual genes affect UCI.

## Conclusion

The reciprocal crossing in longan cultivars Yiduo and Shixia is successful only in one way, i.e., SY, while in the case of YS, it is incompatible. Our results elaborated the differential expression of genes associated with plant-hormone signal transduction, phenylpropanoid biosynthesis, protein processing in the endoplasmic reticulum, and starch and sucrose biosynthesis pathways. The detailed analysis of plant-hormone signaling pathway indicated that JA metabolism and signaling have important roles in UCI. The endogenous JA contents in the YS pollination combination were higher than in SY. In the case of YS, pollen tube growth was slow, and the transcriptome comparison data could be related to it by discussing pollen tube, cell wall, and starch and sucrose metabolism-related transcripts. The candidate genes identified in this study represent key resources for functional studies related to UCI in longan.

## Data Availability Statement

The raw RNA-seq data has been submitted to NCBI SRA and released under the project number PRJNA741615.

## Author Contributions

JW collected the data, and analyzed and drafted the article. DG did the preparation and treatment of experimental materials. JW, DG, DH, and SH revised the manuscript. JC did the microscopic observation. DG and JL guided the experiment, provided funding, and revised the article. All authors have read and approved the final version of this manuscript.

## Conflict of Interest

The authors declare that the research was conducted in the absence of any commercial or financial relationships that could be construed as a potential conflict of interest.

## Publisher’s Note

All claims expressed in this article are solely those of the authors and do not necessarily represent those of their affiliated organizations, or those of the publisher, the editors and the reviewers. Any product that may be evaluated in this article, or claim that may be made by its manufacturer, is not guaranteed or endorsed by the publisher.

## Footnotes

1
www.tridge.com

2
http://www.bioinformatics.babraham.ac.uk/projects/fastqc/

3
www.r-project.org

4
https://www.uniprot.org/uniprot/Q9SIT5

## Abbreviations

12-OPDA: 12-13-epoxylinoleic acid to 12-oxophytodienoate
AA: α -amylase
AOC: allene oxide cyclase
AOS: allene oxide synthase
ARO1: armadillo repeat only 1
BA: β -amylase
BAK: BRI1-associated receptor kinase
BERUCTF: β -fructofuranosidase
BGL: β -glucosidase
BGLs: β -glucosidases
CA3OM: 3-O-methyltransferase
CAD: cinnamyl-alcohol dehydrogenase
CESA: cellulose synthase
CHI: chintinase
CHX15: cation/H(+) antiporter 15
CK: control
CNGC18: cyclic nucleotide-gated channel
COG: clusters of orthologous groups
COI: coronatine-insensitive protein
CPK: calcium dependent protein kinase
DAD: defective in anther dehiscence
DEGs: differentially expressed genes
EG: endoglucanase
ERF: ethylene responsive factor
FDR: false discovery rate
FPKM: fragments per kilobase of transcript per million fragments mapped
GAG: 1,4-alpha-galacturonidase
GH3: glycosylhydrolase family 3
GID: Gibberellin receptor GID
GIP1: glucose-1-phosphate adenylyltransferase
GO: gene ontology
HAP: hours after pollination
IAA: indole-3-acetic acid
INT13: myo-inositol transporter 13
JA: jasmonic acid
JA-Ile: jasmonyl isoleucine
JAR1: jasmonoyl-isoleucine synthetase
JAZ: jasmonate ZIM domain
JMT: jasmonic acid carboxyl methyltransferase
KEGG: Kyoto Encyclopedia of Genes and Genomes
LOX: lipoxigenase
MeJA: methyl jasmonate
MYC2: transcription factor MYC2
OPR: OPDA reductase
PAL: phenylalanine ammonia lyase
PCA: principal component Analysis
PCC: Pearson’s correlation coefficient
PERK: proline-rich receptor-like protein kinases
PLA: phospholipase A1
PME: pectin methylesterase
PMEI: PME-inhibitor
PRP: proline rich protein
RALFL: RALF-like
S: Shixia
SAUR: small auxin up-regulated RNA
SPS: sucrose phosphate synthase
SRK: S-receptor kinase
SS13: strictosidine synthase-like 13
SuSy: sucrose synthase
SYP124: syntaxin-124
TGA: TGA transcription factor
UCI: unilateral cross incompatability
XETs: xyloglucan:xyloglucosyl transferases
XIPs: xylanase inhibitors
Y: Yiduo.
