# Supplementary material for: Investigating the Mechanism of Unilateral Cross Incompatibility in Longan (Dimocarpus longan Lour.) Cultivars (Yiduo × Shixia)
Source: Front Plant Sci. 2022 Feb 11;12:821147. doi: 10.3389/fpls.2021.821147 (PMC8874016; doi:10.3389/fpls.2021.821147)

**Supplementary Figure 1.** Microscopic observation of pollen germination and stigma receptivity.

a. The pollen of Shixia was on the medium germination and growth; b. Germination and growth of Yiduo pollen; c. The stigma receptivity was observed, and the red arrow indicates a large number of bubbles. d. Pistil of Yiduo.

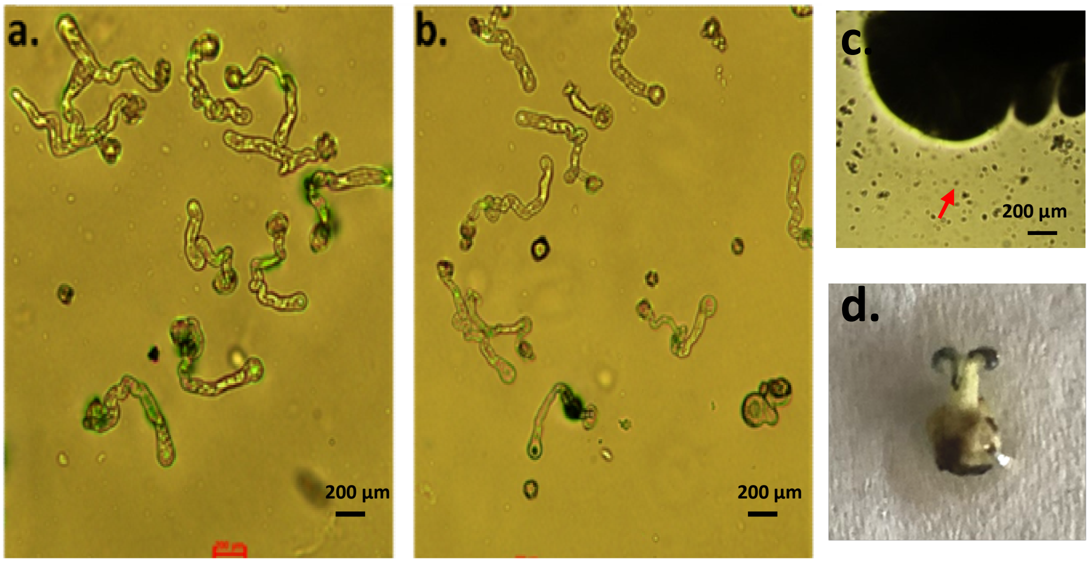

**Supplementary Figure 2.** KEGG enrichment scatter plot showing the pathways in which DEGs were significantly enriched in different treatment combinations i.e., a) CK1 vs YS4, b) CK1 vs YS8, c) CK1 vs YS12, d) CK1 vs YS24, e) CK2 vs SY4, f) CK2 vs SY8, g) CK2 vs SY12, and h) CK2 vs SY24.

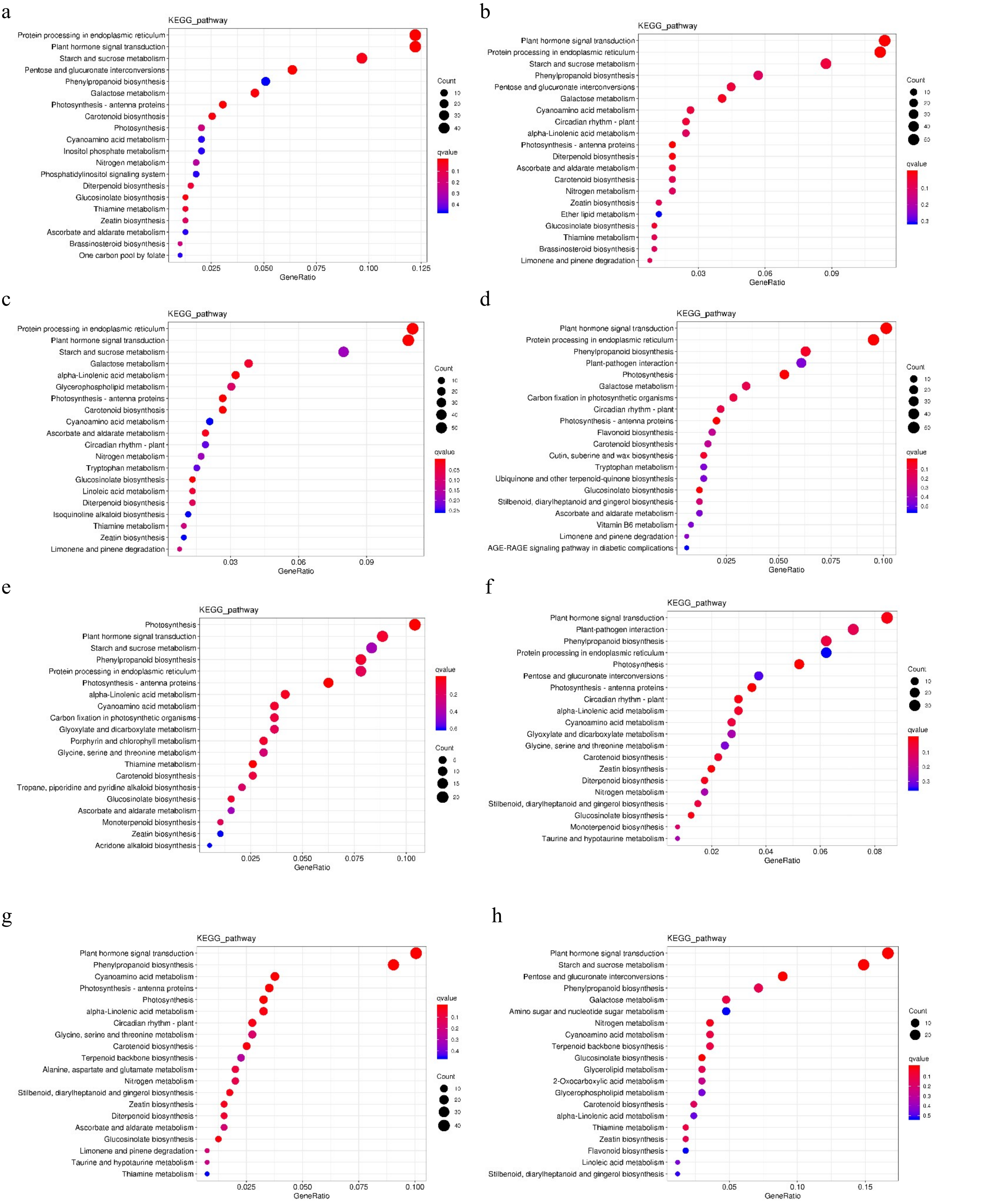

**Supplementary Figure 3.** qRT-PCR analysis of Jasmonic acid metabolism related genes in longan. Y = Yiduo, S = Shixia, CK1 = Yiduo sepal-pistil before pollination, and CK2= Shixia sepal-pistil before pollination. The numbers with treatments represent time after pollination. X-axis represents the cross-pollination combinations and Y-axis represent the relative gene expression. Gene ID with corresponding genes name as follows: *Dlo_022603.1.gene* ( *PLA1* ), *Dlo_012262.1.gene* ( *JAR1* ), *Dlo_024807.1.gene* ( *OPR11* ), *Dlo_031198.1.gene* ( *LOX* ), *Dlo_003095.1.gene* ( *CYP94A2* ), *Dlo_013441.1.gene* ( *CYP94C1* ), *Dlo_010077.1.gene* ( *OPR11*), *Dlo_020248.1.gene* ( *PLA1* ), *Dlo_011584.1.gene* ( *AOC* ), *Dlo_022217.1.gene* ( *OPR11* ), *Dlo_014311.1.gene* ( *PLA1* ), *Dlo_032545.2.gene* (*CYP94C1* ), *Dlo_001507.1.gene* ( *LOX14* ), *Dlo_012184.1.gene* ( *LOX13* ), *Dlo_015582.1.gene* ( *AOS* ), *Dlo_001987.1.gene* ( *JMT* ), *Dlo_006067.1.gene* ( *LOX* ), *Dlo_012185.1.gene* ( *LOX* ), *Dlo_037902.1.gene* ( *LOX* ).

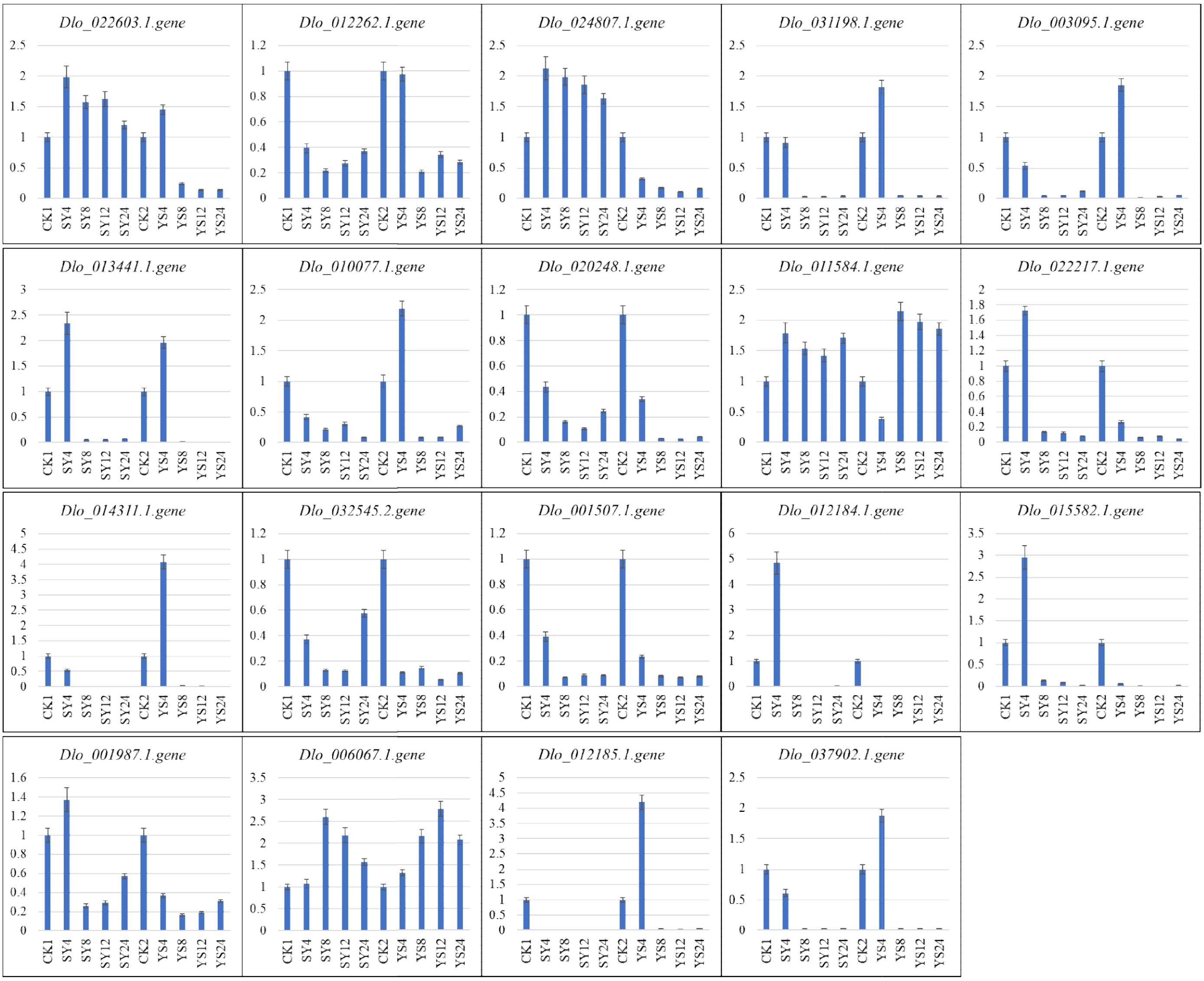

Supplement: Supplementary file 2 [file Data_Sheet_1.PDF]
